# Simulation and Modeling of the Adhesion of *Staphylococcus aureus* onto Inert Surfaces under Fluid Shear Stress

**DOI:** 10.3390/pathogens13070551

**Published:** 2024-06-30

**Authors:** Sarees Shaikh, Abdul Nafay Saleem, Patrick Ymele-Leki

**Affiliations:** 1 Department of Chemical Engineering, Howard University, Washington, DC 20059, USA; sarees.shaikh@bison.howard.edu; 2 Department of Electrical Engineering and Computer Science, Howard University, Washington, DC 20059, USA; abdulnafay.saleem@bison.howard.edu

**Keywords:** bacteria, adhesion, shear stress, *Staphylococcus aureus*, COMSOL, Python, BioFlux

## Abstract

Bacterial adhesion to biotic and abiotic surfaces under fluid shear stress plays a major role in the pathogenesis of infections linked to medical implants and tissues. This study employed an automated BioFlux 200 microfluidic system and video microscopy to conduct real-time adhesion assays, examining the influence of shear stress on adhesion kinetics and spatial distribution of *Staphylococcus aureus* on glass surfaces. The adhesion rate exhibited a non-linear relationship with shear stress, with notable variations at intermediate levels. Empirical adhesion events were simulated with COMSOL Multiphysics^®^ and Python. Overall, COMSOL accurately predicted the experimental trend of higher rates of bacterial adhesion with decreasing shear stress but poorly characterized the plateauing phenomena observed over time. Python provided a robust mathematical representation of the non-linear relationship between cell concentration, shear stress, and time but its polynomial regression approach was not grounded on theoretical physical concepts. These insights, combined with advancements in AI and machine learning, underscore the potential for synergistic computational techniques to enhance our understanding of bacterial adhesion to surfaces, offering a promising avenue for developing novel therapeutic strategies.

## 1. Introduction

*Staphylococcus aureus* is a Gram-positive bacterium that has been recognized as a frequent colonizer of humans and a major opportunistic bacterial pathogen [1,2]. Most pathogens that adhere to biotic or abiotic surfaces subsequently form biofilms, which are structured microbial communities that confer microorganisms with greater resistance to mechanical, physical, or chemical challenges [3,4,5]. These bacteria–host interactions are precursors to bacteremia and invasive infections like endocarditis, peripheral intravenous medical device infections, and septic arthritis [6,7]. Such *S. aureus* infections continue to have a propensity for high morbidity and mortality [7,8].

Bacterial adhesion and subsequent biofilm formation on host tissues and medical implants are critical steps in the pathogenesis of *S. aureus* infections. Since most of these biofilms develop in wet environments, shear stress forces generated by fluid flow have long been recognized to impact bacterial adhesion to surfaces [5,9,10,11,12]. Shear stress is the force per unit area exerted by the fluid moving along the surface of an object, computationally estimated using fluid dynamics models and expressed in units of dynes/cm^2^. This biomechanical force shaped by fluid flow, vessel geometry, and fluid viscosity, is crucial in the physiological context of bacterial adhesion [13].

Physiologically relevant shear stresses in the human vasculature vary with the vessel of interest. For instance, they range from 3 to 5 dyn/cm^2^ in brachial arteries and from 1 to 6 dyn/cm^2^ in most veins [14,15]. In this study, the kinetics of *S. aureus* cells’ adhesion to abiotic surfaces were investigated under varied fluid shear forces. The use of inert glass surfaces in this study provides a simple and controlled methodology to examine bacterial adhesion, allowing a focus on understanding the fundamental principles of adhesion without the interference of surface chemistry variations [16,17]. Cell adhesion assays were performed in relevant hydrodynamic conditions with a BioFlux 200 microfluidic system. Theoretical and empirical studies have shown that cell spatial distribution plays a critical role in the physiological properties of bacteria in their natural milieus [18,19]; thus, the effect of shear stress on the overall organization and pattern of bacterial adhesion in the microfluidic system was investigated using MATLAB and COMSOL Multiphysics^®^ software. Given the propensity of COMSOL Mutliphysics^®^, a well-known commercial fine element modeling package, to serve as a tool of choice for modeling physiological transport phenomena [20,21], the aim of this study was to develop a simple COMSOL multiphysics model to evaluate the adhesion kinetics of free-floating bacteria in hydrodynamic milieus.

To complement these computational approaches, Python was employed to model the relationship between shear stress, time, and bacterial surface concentration using polynomial regression. This modeling aimed to capture the non-linear correlations within our data and generate a robust mathematical representation of the adhesion kinetics observed in experimental assays. Ultimately, the insights gained from such studies could inform the development of novel therapeutic approaches to prevent or treat *S. aureus* infections, particularly those associated with medical devices or implanted tissues.

## 2. Materials and Methods

### 2.1. Bacterial Strains and Cultures

A *Staphylococcus aureus* Phillips strain served as the model organism for this study. This strain was originally isolated from a patient with osteomyelitis and was chosen for its documented adhesion capabilities and proficiency to form biofilms under hydrodynamic conditions [22,23,24]. Bacterial glycerol stocks stored at −80 °C were revived in 50 mL of Tryptic Soy Broth without dextrose (TSB; Bacto^®^, BD; Franklin Lakes, NJ, USA), in a shaking flask incubator at 37 °C with continuous rotation, as previously described [23,24,25]. As applicable, cells were diluted with phosphate-buffered saline (PBS; 138 mM NaCl, 2.7 mM KCl [pH 7.4]) to achieve a bacterial concentration of $1\times 10^{7}$ cells/mL—as determined with a cell counter (Beckman Coulter Multisizer 4). As previously described, PBS hindered further bacterial growth and ensured a controlled, physiologically relevant environment devoid of nutrients that could interfere with the adhesion process [23,24,25,26,27].

### 2.2. Adhesion Assay under Hydrodynamic Conditions

Real-time adhesion studies of *S. aureus* on glass surfaces were performed using a BioFlux 200 microfluidic system as described in previously published studies [28], with minor adjustments made to the protocol. Briefly, 1 mL of bacteria suspension, diluted to 1 × 10^7^ cells/mL in phosphate buffer saline (PBS) at 37 °C, was pipetted to the BioFlux plate’s input well. To mimic physiologically and dynamically relevant conditions, adhesion assays were investigated at wall shear forces ranging from 1- to 5 dyn/cm^2^ through the pressure interface of the BioFlux system. The flow system was connected to a Zeiss AXIO Observer microscope for image acquisition.

The automated microscope and the Zen Pro software were used to capture images from the BioFlux plate channel. For each assay, three images were systematically acquired along the flow channel length at 5-minute intervals during a 1-hour experimental run. These images were strategically positioned at the center of the channel. Each experimental run was conducted in triplicates: three channels were run simultaneously for each shear setting on any given plate; three images were captured at each time point; and the same conditions were repeated for at least three plates in independent assays. This process resulted in a total of 585 images generated for the analysis of five wall shear conditions.

### 2.3. Determination of Bacteria Surface Concentrations, Spatial Analysis and Adhesion Rates

Once the images were captured, they were analyzed using the OpenCFU 3.9.0 software. OpenCFU was used to automate the detection and quantification of bacterial colonies, assigning each cell a unique X and Y coordinate. The output, containing the coordinates of each detected cell, was organized into an Excel sheet. This process was iterated for all images generated.

The surface concentrations (in cells/µm^2^) of bacteria cells are determined by taking the number of cells detected from each Excel file and dividing by the area of the image captured. The surface concentrations are then plotted against time for all shear stress values. The process is repeated for at least three experimental replicates per assay plate; and each plate experiment is repeated multiple times, and the results are averaged.

To determine the maximum adhesion rates of bacteria cells, the slope of the surface concentration vs. time graph for the first 20 min was calculated. The maximum adhesion rates were plotted against the shear stress values as a bar graph. The process was repeated for all experimental iterations and averaged.

A MATLAB script written for this study was used to process spatial data from the Excel file generated using OpenCFU for each image of the experiment (see Supplementary Materials). The script imports the data into a table that lists the individual X and Y coordinate matrices of each bacteria cell. These coordinates are then combined into a single matrix, facilitating subsequent distance calculations. Using the “pdist” function with the Euclidean metric, the script computes distances between all pairs of points, resulting in a distance matrix. The average distance between points, calculated as the mean of all pairwise distances between *S. aureus* cells, provides a broad measure of how the cells are distributed across the image. This metric is essential for understanding the overall spatial arrangement and dispersion of cells. Additionally, the script determines the non-zero distances between the closest pairs of points on the image, constructing a vector of these values. The mean of these distances between neighboring points is computed and reported. This analysis offers insight into both the overall spread of bacterial cells and separations between individual adhered cells. The final output includes the mean spatial distribution and mean spatial clustering, both presented in micrometers, and is repeated for all 585 images.

### 2.4. Multiphysics Simulation in COMSOL

The COMSOL Multiphysics^®^ version 6.1 application was used to develop a two-dimensional multiphysics model that would simulate the adhesion of *S. aureus* cells. The choice of a two-dimensional channel simplified the computational analysis while retaining biological relevance and ensuring experimental relevance. The dimensions of the analytical channels were 400 μm by 70 μm (width × height) to mimic the dimensions of the BioFlux well plates. Similarly, the flow parameters used for the simulations were taken from the BioFlux 200 system and from the literature, when applicable. The applied wall shear stress levels (*τ*) ranged from 1 to 5 dyn/cm^2^ to simulate different fluid flow conditions that the bacteria may encounter in vivo [29]. The density of the fluid in the system (*ρ*) was held constant at 1000 kg/m^3^, which represented the approximate density of phosphate-buffer saline [30]. The viscosity of the fluid (*η*) was calculated from *γ*, the fluid shear rate, with the following equation:

$$\eta =\frac{\tau }{\gamma }$$

Values for *τ* and *γ* are obtained from BioFlux 200. Bacteria cells were introduced as solid particles with 1 μm diameter, to model the average size of *S. aureus* cells, with flow-determined release times. Other relevant parameters are presented in Table 1.

To simulate the interactions between the bacteria and the glass slide of the microfluidic system, two channel wall scenarios were experimentally simulated. In the first scenario, the “no-slip and non-leaking” conditions are applied to all walls of the channel, where walls 1 and 4 act as the inlet and outlet, respectively. This scenario better reproduces the hydrodynamic conditions of the fluid flow within the BioFlux microplates. In the second scenario, walls 2 and 3 now act as “leaking” walls with leaking velocity equal to 10% of inlet velocity. All walls in this scenario still have “no-slip” conditions, while walls 1 and 4 still act as inlet and outlet, respectively. This scenario does not generate a fully developed laminar flow but better integrates the phenomena of bacterial adhesion—without the outright insertion of an adhesion kinetics factor at the channel’s wall.

### 2.5. Data-Driven Modeling in Python

Python was used to model the relationship between shear stress, time, and surface concentration using polynomial regression (see Supplementary Materials). To capture the non-linear correlations within our data, the dataset was first structured into a Pandas data frame. This enabled efficient handling and manipulation of the data throughout the modeling process. Then, a polynomial regression model was set up using scikit-learn, a versatile open-source Python library module for machine learning, with a degree of 3, as previously described [31].

Once the model was set up, the polynomial regression model was trained on the dataset to learn the underlying relationships between shear stress, time, and surface concentration. During the training process, the model adjusted its parameters to minimize prediction errors, effectively capturing the complex dynamics of the system. After training, the coefficients and intercept of the fitted model were extracted to construct the polynomial equation representing the model.

### 2.6. Statistical Analysis

Unless otherwise specified, the data reported are the averages of the mean value of three or more experimental runs. Error bars represent the standard deviations of the means, and statistical significance was calculated using the single-factor analysis of variance technique at a 95% confidence interval (equivalent to *p* < 0.05).

To ascertain the accuracy of the polynomial regression models in Python, the R-squared value was computed, and a residual plot (see Supplementary Materials) was generated to visualize the residuals against the predicted values.

## 3. Results

### 3.1. Two-Dimensional Spatial Distributions of Adhered Bacteria Cells Were Independent of Hydrodynamic Shear Stress

**Figure 1:**
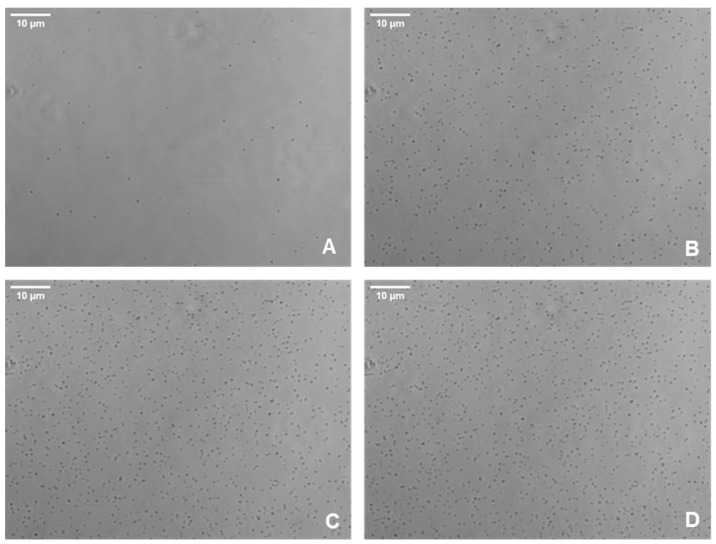
Representative phase contrast images of *S. aureus* cells adhering to the glass surface of the Bioflux microfluidic system. Images of bacteria adhering to surfaces within the microfluidic system were captured using an AXIO Observer microscope at wall shear forces of 2 dyn/cm^2^ at times 0 (**A**), 20 (**B**), 40 (**C**), and 60 (**D**)—which correspond to the start of the assay and three subsequent twenty-minute intervals.

**Figure 2:**
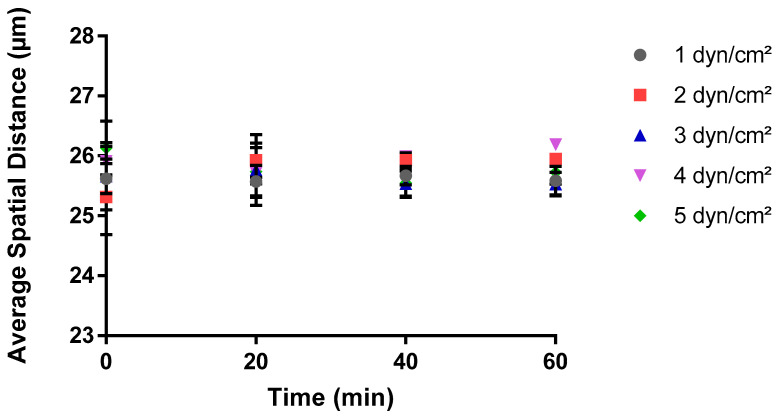
Impact of shear stress on the average spatial distance in the Bioflux microfluidic system. A MATLAB script was used to calculate the average distance between all the adhered cells over time at each shear stress value. Three experimental replicates were performed on separate days, with a total of 180 images generated for analysis. These images represented cell adhesion at twenty-minute intervals for triplicate areas in each experiment. The data are the average of all experimental replicates at each shear stress and time point.

Images of bacteria adhering to surfaces within the microfluidic system were captured using an AXIO Observer microscope and the Zen Pro software, under varying wall shear forces from 1 to 5 dyn/cm^2^ (Figure 1). Then, the spatial distribution of bacteria was investigated by monitoring the mean spatial distribution between cells and the average distance between neighboring cells at varying wall shear stresses. The mean spatial distribution between cells depicted a steady trend when subjected to various shear stress levels over a 60-minute period during BioFlux assays (Figure 2). Remarkably, the average distance remained consistent at approximately 26 µm across all shear stress conditions, showing no significant change as time progressed. This uniformity suggested that the average spatial distribution of cells on the surface was not significantly influenced by the different shear stresses applied within the range of 1 to 5 dyn/cm^2^. This constant spatial distance implied a homogeneity in cell distribution post-adhesion, indicating that once cells adhere to the surface, their spatial organizations did not dynamically change with time under the shear stress conditions tested.

**Figure 3:**
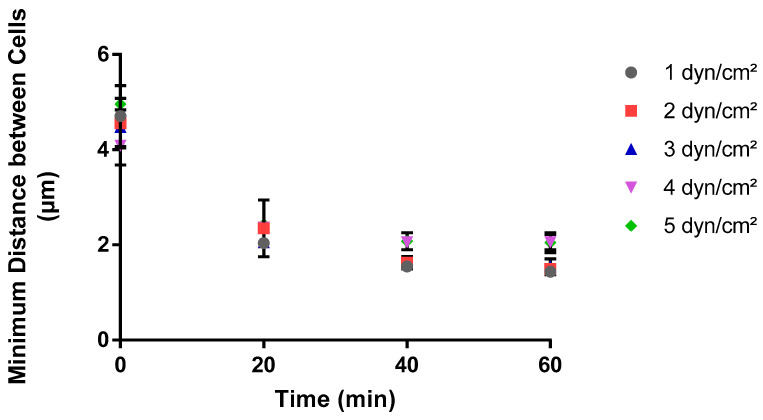
Impact of shear stress on the minimum average distance between adhered cells over time. A MATLAB script was used to calculate the average distance between the closest adhered cells over time at each shear stress value. Three experimental replicates were performed on separate days, with a total of 180 images generated for analysis. These images represented cell adhesion at twenty-minute intervals for triplicate areas in each experiment. The data are the average of all experimental replicates at each shear stress and time point.

In contrast, the average distance between neighboring cells displayed a decreasing trend under various shear stress conditions over a 60-minute timeframe (Figure 3). Initially, at time zero, the average distance between neighboring cells was approximately the same for all shear stresses, at about 5 µm. As time advanced, this distance decreased sharply within the first 10 to 20 min, indicating that cells were more closely packed as their surface concentration increased over time.

The curves for all shear stress levels converged to a smaller distance after the initial sharp decline, suggesting that towards the end of the 60-minute observation, the cells adhered to the surface in a random and closely packed manner. Convergence to a minimum plateau value at different times for different shear stresses was observed, with the higher shear stresses (4 and 5 dyn/cm^2^) reaching a plateau earlier than the lower shear stresses. By the end of the observation period, higher shear stresses (4 and 5 dyn/cm^2^) converge to a 33% higher mean distance than lower shear stresses (1–3 dyn/cm^2^). This trend explains how as the shear stress increases the cells are more loosely packed and the spatial clustering between the cells is lower.

### 3.2. Bacterial Surface Coverage Decreased with Increasing Wall Shear Forces

**Figure 4:**
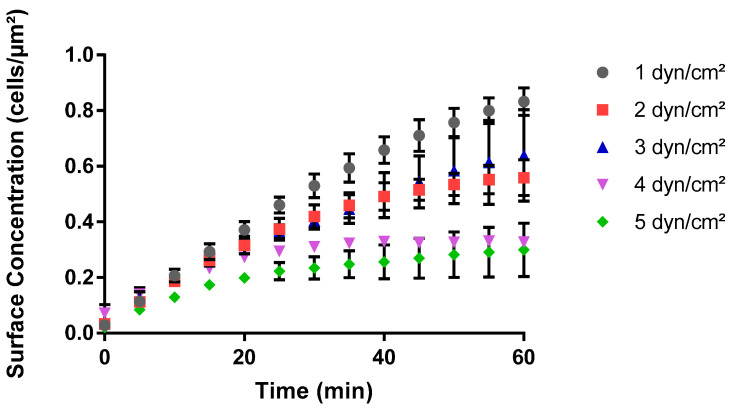
Impact of shear stress on the surface concentration of cells in BioFlux system. *S. aureus* cells in PBS suspension at 37 °C flowed through the microplates at wall shear stress values between 1 and 5 dyn/cm^2^. The data show how the number of cells that adhered per unit area, i.e., surface concentration of cells, varies over 60 min. Images of adhered cells were captured at five-minute intervals for triplicate areas in each experiment, and three experimental replicates were performed on separate days.

The process of bacterial adhesion to a surface, a precursor to further colonization, biofouling, or infectious mechanisms, is influenced by several environmental factors in hydrodynamic milieus. Fluid shear stress is one such factor. Bacterial cell adhesion under varying fluid shear stresses in BioFlux assays revealed a non-linear relationship between wall shear stress levels and surface concentration of cells over a 60-minute period. The surface concentration of cells increased over time, at a decreasing rate, for all shear stress levels (Figure 4). At 1 dyn/cm^2^, there was an initial steep ascent followed by a plateau at a final surface concentration. Whereas at a wall stress of 5 dyn/cm^2^, the surface concentration of bacterial cells increased moderately until it plateaued at a 2.78-times-lesser surface concentration level than at 1 dyn/cm^2^. Overall, the final surface concentration showed a declining trend as wall shear stress increased, although an outlier was observed at shear stress 3 dyn/cm^2^. The bacterial surface concentration at 3 dyn/cm^2^ was not only greater than that at 2 dyn/cm^2^ from the outset but also continued to diverge further over time (Figure 4). This observation suggested that the cells experienced a more favorable adhesion environment at 3 dyn/cm^2^ compared to 2 dyn/cm^2^. This outlier suggested that cell adhesion in BioFlux assays was not solely governed by the magnitude of the shear stress but also by other factors that could be influencing cell adhesion kinetics at specific shear stress levels.

**Figure 5:**
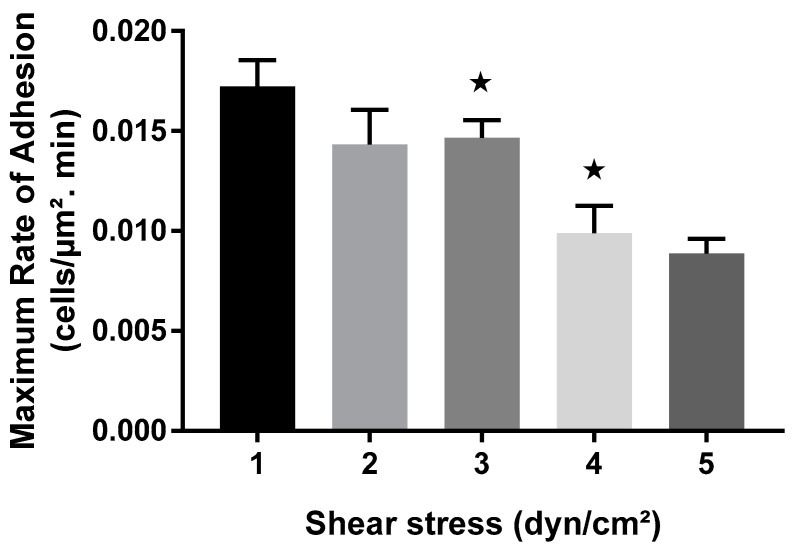
Impact of shear stress on the maximum rate of adhesion in BioFlux system. Data show how the maximum rate of adhesion varies over wall shear stress values ranging from 1- to 5-dyn/cm^2^. Data represent average values from three experimental replicates, with triplicate runs for each experiment. Stars represent statistical significance (*p* < 0.05) from *p*-value test between the two columns.

A similar pattern was observed while investigating the impact of wall shear stress on the maximum rate of adhesion of bacterial cells (Figure 5). The maximum rate of adhesion was observed in the first 20 min for all shear conditions investigated (Figure 4). Similar to the surface coverage data, the kinetics data displayed a non-linear relationship between shear stress and the maximum rate of cell adhesion (Figure 5). The maximum adhesion rate was highest at the lowest wall shear stress of 1 dyn/cm^2^. Overall, a decrease of almost 2-fold in the rate of adhesion was observed as the shear stress level increased from 1 to 5 dyn/cm^2^. For instance, as the shear stress increased from 1 dyn/cm^2^ to 2 dyn/cm^2^, there was a 16% reduction in the adhesion rate. However, an unexpected increase was observed at 3 dyn/cm^2^. The adhesion rate at 3 dyn/cm^2^ demonstrated a local peak that featured higher values than what was observed at both 2 dyn/cm^2^ and 4 dyn/cm^2^ (Figure 5) and was consistent with the surface coverage data (Figure 2). Taken together, these results could have implications for understanding cellular responses to mechanical forces in various biological and biomedical applications.

### 3.3. Bacterial Adhesion May Be Simulated in COMSOL with Leaking Wall Boundary Conditions

**Figure 6:**
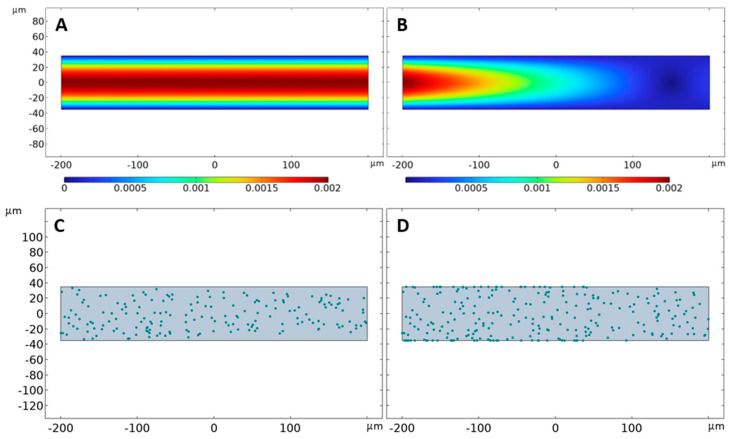
COMSOL simulation scenarios for bacterial cell adhesion under hydrodynamic milieus. A microfluidic channel was simulated in COMSOL, using the parameters described in Table 1. Two scenarios were investigated: (**A**,**C**) the channel had fixed walls with no slip, and wall 4 served as the only outlet; and (**B**,**D**) the channel had fixed walls with no slip, wall 4 still served as the main outlet but walls 2 and 3 acted as leaking walls. *S. aureus* cells were represented by green spheres of 1 µm diameter. Images show representative flow and adhesion profiles at the wall shear stress value of 1 dyn/cm^2^ captured at time *t* = 5 min during a sixty-minute run.

Adhesion kinetics of free-floating bacteria in hydrodynamic conditions were theoretically simulated with the COMSOL Multiphysics^®^ software using two distinct wall boundary conditions. In the first scenario, the COMSOL setup used the flow parameters defined in Table 1 coupled with non-leaking, no-slip wall boundary conditions. The channel with the dimensions of 400 µm by 70 µm mimicked the boundary wall and flow conditions of the BioFlux 200 microfluidic environment. All the walls of the channel featured a “no-slip” boundary condition. Vertical walls at the extremes of the channel act as the inlet and outlet. The data showed a fully developed velocity profile with maximum velocity towards the center of the channel and decreasing velocity as we move closer to the walls (Figure 6A). The velocity profile corroborated the presence of a fully developed laminar flow in the Bioflux microfluidic channel during in vitro adhesion experiments (Figure 3 and Figure 4). COMSOL simulations also accounted for *S. aureus* cells that could be seen as 1 µm diameter circles (Figure 6C) floating in the channel and moving with the fluid. However, no bacterial adhesion to the walls of the microfluidic channel was observed during the hour-long fluid flow simulation under these in silico conditions. Instead, the bacteria cells were observed to be sliding off the wall in the direction of flow. Since there was no adhesion observed under the above COMSOL scenario, the simulation setup was altered to exhibit relevant adhesion of cells.

In the second COMSOL scenario, relevant flow channel walls were modified to retain their “no-slip” boundary conditions but incorporate leaking surface properties. In this case, the horizontal walls acted as leaking walls. While the vertical walls serving as the main inlet and outlet of the channel remained unchanged, the horizontal walls now included a leaking velocity factor that represented 10% of the maximum velocity in the channel. This setup resulted in a non-laminar velocity profile but exhibited significant cell adhesion to the channel walls (Figure 6B,D). Taken together, these data suggested that, although trade-offs were made in the simulation of fluid flow properties, leaking wall boundary conditions were better suited for the simulation of bacterial adhesion to surfaces under hydrodynamic conditions in COMSOL; thus, the COMSOL scenario including leaking walls was replicated to investigate the impact of wall fluid shear stress on bacterial adhesion.

### 3.4. COMSOL Simulations Corroborated Results from Microfluidics Studies

**Figure 7:**
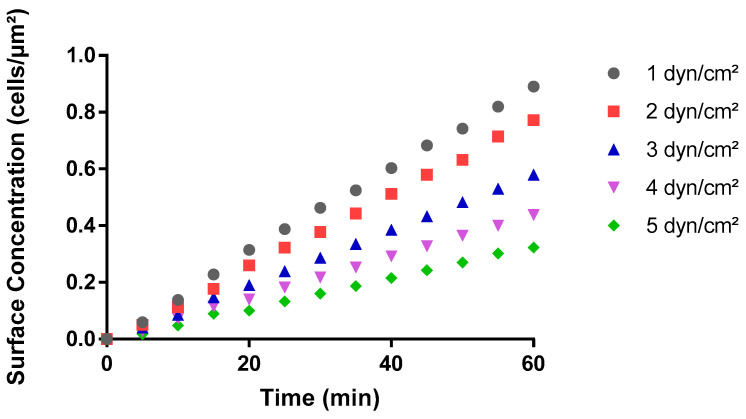
Impact of shear stress on the surface concentration of bacteria cells in COMSOL. The graph shows how the number of cells adhered per unit area (i.e., the surface concentration) varies with respect to time, in a 400 by 70 μm rectangular channel. These data are a result of simulating the adhesion of solids with 1 µm diameter in presence of a 10% leaking wall velocity at different wall shear stress values ranging from 1 to 5 dyn/cm^2^.

**Figure 8:**
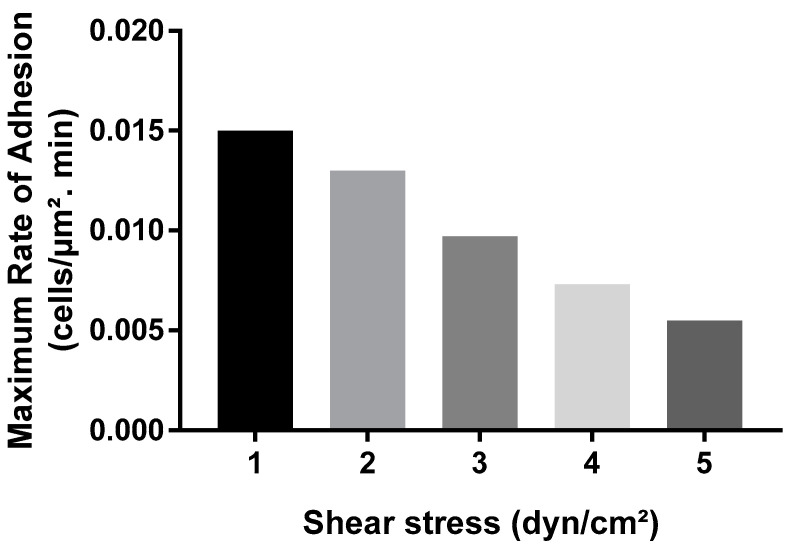
Impact of shear stress on the maximum rate of bacterial adhesion in COMSOL. The graph shows the trend of adhesion rates for the first 20-min interval of the COMSOL simulation at different shear stress values ranging from 1 to 5 dyn/cm^2^.

Overall, COMSOL data reproduced the trends previously observed with bacterial adhesion in BioFlux microwell plates (Figure 4 and Figure 5). As hydrodynamic shear forces increased, the simulations predicted that the cell surface concentration at any given time would decrease accordingly (Figure 7). The final surface concentration across all shear stress levels revealed a trend where higher shear stress resulted in lower cell adhesion, as observed in BioFlux experiments (Figure 4). Overall, the COMSOL simulations supported the notion of a robust inverse relationship between shear stress and cell adhesion, suggesting that higher shear stresses may inhibit the adhesion of cells to surfaces (Figure 7 and Figure 8); however, in contrast to microfluidics experiments that revealed plateauing concentration values over time, the simulations projected a linear increase in cell surface concentration at all shear conditions (Figure 7). The theoretical data from COMSOL also predicted a linear reduction in the rate of adhesion of bacterial cells with increasing fluid shear stress (Figure 8). Taken together, these data suggest that while the theoretical results could predict the general direction of change and overall magnitude of relevant parameters, they did not account for all the biological and dynamical factors at play in situ at the cell–surface boundary of the microfluidic system.

### 3.5. Python Modeling Reproduced the Non-Linear Relationship between Bacterial Adhesion and Wall Shear Stress

Python modeling provided a mathematical representation of the non-linear relationship between bacteria surface concentration (*C*), wall shear stress (*τ*), and time (*t*). The model employed a polynomial regression approach to fit the observed data. The resulting polynomial equation describing the surface concentration, *C*(*t*,*τ*), in terms of time, *t*, and wall shear stress, *τ*, is as follows:

*C*(*t*,*τ*) = (5.3330 × 10^−2^ − 8.4290 × 10^−2^
*τ* + 4.6928 × 10^−2^
*τ*^2^ − 6.185 × 10^−3^
*τ*^3^) + (2.6465 × 10^−2^ − 4.278 × 10^−3^
*τ* + 2.46 × 10^−4^
*τ*^2^)*t* + (−2.11 × 10^−4^ + 7 × 10^−6^
*τ*)*t*^2^ + 1 × 10^−6^
*t*^3^, (1)

**Figure 9:**
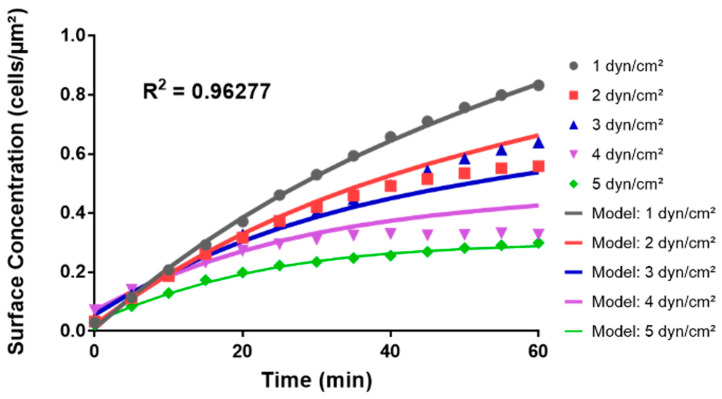
Comparison of the Python theoretical model to empirical data in the microfluidic system. The graph juxtaposes the curves predicted from the Python polynomial model in Equation (1) (“Model:”) with the averages of cell surface concentrations from the adhesion assays in the microfluidic system at wall shear stress values ranging from 1 to 5 dyn/cm^2^.

This model captured the non-linear relationship between wall shear stress and surface concentration of cells over a 60-minute period (Figure 9). For instance, at 1 dyn/cm^2^, the model predicted an initial steep increase in surface concentration, followed by a plateau, aligning well with the experimental observations; conversely, at 5 dyn/cm^2^, the model predicted a more gradual increase in surface concentration, eventually plateauing at a level 2.89 times lower than at 1 dyn/cm^2^, consistent with the experimental trend (Figure 4 and Figure 9). The model had an R^2^ value of 0.96277, indicating an overall good fit with the data which was skewed at intermediate shear stress conditions by the outlier trend of adhesion observed at 3 dyn/cm^2^ (Figure 4).

Thus, to further validate the polynomial regression approach and confirm its accuracy in representing the underlying dependence of cell surface concentration on time and shear stress, a theoretical model that excluded the experimental data for shear stress 3 dyn/cm^2^ was generated. This model gave the following equation for surface concentration, *C*(*t*,*τ*):

*C*(*t*,*τ*) = (8.794 × 10^−3^ − 3.9318 × 10^−2^
*τ* + 3.9432 × 10^−2^
*τ*^2^ − 6.185 × 10^−3^
*τ*^3^) + (3.0093 × 10^−2^ – 8.027 × 10^−3^
*τ* + 8.71 × 10^−4^
*τ*^2^)*t* + (−2.06 × 10^−4^ + 7 × 10^−6^
*τ*)*t*^2^ + 1 × 10^−6^
*t*^3^, (2)

**Figure 10:**
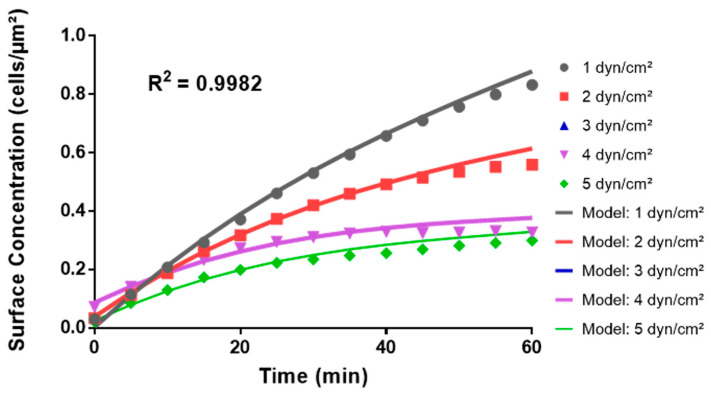
Comparison of the Python theoretical model to empirical data in the microfluidic system. The graph juxtaposes the curves predicted from the Python polynomial model in Equation (2) (“Model:”) with the averages of cell surface concentrations from the adhesion assays in the microfluidic system at wall shear stress values of 1-, 2-, 4-, and 5 dyn/cm^2^.

This adjusted model (Equation (2)) generated a plot (Figure 10) with an R^2^ value of 0.9982, suggesting that ignoring the outlier at shear stress 3 dyn/cm^2^ yielded a more accurate mathematical representation of the change in bacteria surface concentration with time at wall shear stresses of 1, 2-, 4-, and 5 dyn/cm^2^ under the investigated experimental conditions. Taken together, these data suggest that the Python models developed through machine learning polynomial regression accurately reflect the observed empirical trends and capture the complex interplay between shear stress and bacterial adhesion over time.

## 4. Discussion

Bacterial adhesion to biotic and abiotic surfaces plays a pivotal role in the mechanisms of microbial colonization and biofilm formation [32]. Depending on the circumstances, initial attachment to surfaces may lead to biofouling, biocorrosion, impeded performance of industrial and biomedical devices, or the survival and resilience of dangerous pathogens [33]. Thus, understanding the rules governing these mechanisms or developing novel antimicrobial approaches starts with investigating the biophysics of bacterial adhesion in their hydrodynamic milieus. This study explored the intricacies of bacterial adhesion dynamics under various hydrodynamic forces, leveraging both experimental bioassays and advanced computational simulations. Specifically, the impact of wall shear stress on the adhesion and spatial distribution of bacteria cells was examined using BioFlux microfluidics assays and COMSOL Multiphysics^®^ simulations.

Taken together, the data suggested that the two-dimensional spatial distributions of adhered bacteria cells were largely independent of varying shear stresses, maintaining a consistent average distance between cells across all conditions. Interestingly, while the overall spatial distribution remained steady under varying shear stresses, the distance between neighboring cells decreased over time due to ongoing cell accumulation on the surfaces in fluid flow conditions. The BioFlux assays highlighted a non-linear relationship between shear stress and bacteria surface concentration, with an unexpected increase in adhesion rates at the intermediary shear stress of 3 dyn/cm^2^, suggesting that factors beyond sheer mechanical forces are at play in bacterial adhesion. For the most part, COMSOL simulations further supported BioFlux results, indicating a clear decreasing trend in the maximum rate of adhesion as shear stress increased. The consistent findings across both experimental and simulation approaches underscore the complex nature of cell adhesion under fluid shear stress and hint at the existence of non-specific interactions that promote adhesion under fluid flow [34].

This study further highlighted current limitations in the predictive capability of the Multiphysics software in simulating dynamic biological interactions. Embedded COMSOL simulation functions, though robust, fell short of accounting for the complex biological and physical interactions at play between bacteria cells and abiotic nonporous surfaces under fluid flow. As highlighted in previous studies, the Multiphysics software does not incorporate all fluid–structure interactions and does not model the three-dimensional deformations of flexible biological structures under fluid forces [35]. However, the introduction of leaking wall conditions in COMSOL simulations to mimic physiologically relevant environments suggested a potential avenue for software enhancement, with better characterization of non-linear and non-specific interactions. For instance, the leaking walls scenario in COMSOL may depict the physiologically relevant case of a biotic or abiotic surface that allows for the diffusion of materials, as is the case for blood vessel permeability [36,37,38].

On the other hand, Python modeling provided a robust mathematical representation of the non-linear relationship between bacterial surface concentration, wall shear stress, and time. The polynomial regression approach used in this study accurately captured the observed empirical trends and the complex interplay between shear stress and bacterial adhesion over time. Such models are crucial for mathematically predicting complex relationships, offering insights that can guide experimental design and therapeutic strategies. However, while COMSOL can predict effects on surface concentration based on design parameters such as channel shapes, gravity, and changes in fluid properties, the Python model is limited to mathematical predictability based solely on the independent variables of shear stress and time. Even though the Python model showed an excellent fit with the empirical data, as corroborated by its high R^2^ value, it does not reveal the causal mechanisms underlying the bacterial adhesion phenomena observed in the microfluidics system.

This distinction underscores the complementary nature of these modeling approaches, with Python providing statistical correlations and COMSOL offering broader causative scientific understanding, owing to its inherent restriction to well-defined scientific variables in its simulations. Other studies have pointed out that regression models often fail to account for the intricate, multi-scale interactions governing biological phenomena, especially when it comes to understanding the dynamic behavior of biological systems [39]. Several experimental techniques and assays for investigating the biophysics of bacterial adhesions and infections have been developed and adopted by the scientific community [32,40,41]. Similar consensuses remain to be developed for the in silico studies of these biophysical events; however, in silico simulations have become an essential tool for the study of biological phenomena [42,43].

On a broader scale, computer simulations have played significant roles in the design or fabrication of microfluidic channels for biomedical applications [44]. On a microscale, they may help design new drugs or better capture the complexities of dynamic biological phenomena, like the impedance of single HeLa cells [45,46]. With the advent of AI and machine learning tools, the surge and impact of in silico models are poised to become more useful, accurate, and prominent than ever. The promises of multiphysics simulation tools are undeniably more overarching than in vitro assays; however, lessons learned from comparative studies such as this one point to the need for greater synergy between empirical findings and computational modeling approaches, particularly those guided by well-defined scientific variables.

## Figures and Tables

**Table 1:** Relevant biological and physical properties of the simulation system at different shear stresses.

Symbol	Value					Unit	Description
*t*	1	2	3	4	5	dyn/cm^2^	Shear Stress
*U*_0_	1337.959	2675.736	4002.267	5340.136	6678.005	μm/s	initial velocity
*P*_0_	0.4	0.8	1.19	1.59	1.99	psi	inlet pressure

*U*_0_ is the initial velocity of fluid flow through the system, calculated using *U*_0_ = Q/A, where Q is volumetric flowrate from BioFlux 200 and A is the cross-sectional area of the channel. *P*_0_ is the pressure at the system inlet, also determined from the BioFlux 200 system.

## Data Availability

All raw data will be made available by the corresponding author upon request. The computational models performed on COMSOL software are not publicly available because the models do not stand alone. All key analyses of the computational data are presented in the manuscript.

## Supplementary Materials

The following supporting information can be downloaded at: https://www.mdpi.com/article/10.3390/pathogens13070551/s1, S1: Matlab code; S2: Python code; S3: Residual plots.
