# Supplementary material for: Simulation and Modeling of the Adhesion of Staphylococcus aureus onto Inert Surfaces under Fluid Shear Stress
Source: Pathogens. 2024 Jun 30;13(7):551. doi: 10.3390/pathogens13070551 (PMC11280353; doi:10.3390/pathogens13070551)

# Simulation and Modeling of the Adhesion of *Staphylococcus aureus* onto Inert Surfaces under Fluid Shear Stress


**Sarees Shaikh [1], Abdul N. Saleem [2], Patrick Ymele-Leki [1,*]**

[1] Department of Chemical Engineering, Howard University, Washington, DC 20059
[2] Department of Electrical Engineering and Computer Science, Howard University, Washington, DC 20059
* Correspondence: patrick.ymeleleki@howard.edu


## Supplementary Materials

**S1. MATLAB Code**: The following code was used to calculate average pairwise distances between all adhered cells and the average non-zero distances between neighboring cells. The code also generates a histogram to show the frequency distribution of distances between all adhered cells.

```
clc
clear all
clc
% Import the data from the Excel file into a table

filename = 'Experiment-15_s3t01.xlsx'
data = readtable(filename);

% Extract the x and y coordinats into separate matrices
x = data.X;
y = data.Y;

% Combine the x and y coordinates into a single matrix
points = [x y];


% Calculate the distances between all pairs of points using the pdist function
distances = squareform(pdist(points, 'euclidean'));

% The resulting matrix 'distances' will contain the distances between all pairs of
points

average_distance = mean(distances(:))* (7/172);

% Initialize a vector to store the minimum non-zero distances
min_nonzero_distances = zeros(1, size(distances, 2));

% Loop over the columns of the distances matrix
for i = 1:size(distances, 2)
    % Extract the current column
    current_column = distances(:,i);
```

```
    % Find the minimum non-zero distance for the current column
    min_nonzero_distance = min(current_column(current_column > 0));

    % Store the result in the min_nonzero_distances vector
    min_nonzero_distances(i) = min_nonzero_distance;
end

% The resulting vector 'min_nonzero_distances' will contain the minimum non-zero
distance for each pair of points

average_min_distance = mean(min_nonzero_distances(:)) * (7/172);
fprintf('Average distance between the points (in µm) is : %f\n',average_distance);
fprintf('Average min distance between the points (in µm) is :
%f\n',average_min_distance);

% Assuming 'distances' contains the computed distances
non_zero_distances = distances(distances > 0) * (7/172); % Exclude zero distances and
apply unit conversion

% Create the histogram
h = histogram(non_zero_distances);

% Get the histogram data
binCounts = h.Values; % Number of points in each bin
binEdges = h.BinEdges; % Edges of the bins

% Since binEdges has one more element than binCounts, calculate bin centers or use
the first n-1 edges
binCenters = (binEdges(1:end-1) + binEdges(2:end)) / 2;

% Create a table with the histogram data
histogramData = table(binCenters', binCounts', 'VariableNames', {'BinCenter',
'Count'});

% Save the histogram data to an Excel file
writetable(histogramData, filename);

disp('Histogram data saved to HistogramData.xlsx');
```

**S2. Python Code**: The following code was used to generate a polynomial regression fit for the data and compute an equation for surface concentration, C (t, τ), in terms of time, t, and shear stress, τ. The code also computes the R-squared value for the generated model.

```
import numpy as np

import pandas as pd

import matplotlib.pyplot as plt

from sklearn.preprocessing import PolynomialFeatures

from sklearn.linear_model import LinearRegression


# Provided data

data = pd.DataFrame({

    'shear_stress': np.repeat([1, 2, 3, 4, 5], 13),

    'time': np.tile([0, 5, 10, 15, 20, 25, 30, 35, 40, 45, 50, 55, 60], 5),

    'surface_concentration': [

        0.02955647344, 0.114368681, 0.2072670936, 0.2925440257, 0.3710359811,
0.4606348502, 0.5294605376, 0.5939642878, 0.6578638963, 0.7104242288, 0.7572219784,
0.7994654286, 0.8320890833,

        0.03383193815, 0.1126259644, 0.1871212898, 0.2624763553, 0.3173370737,
0.3733336367, 0.4199249906, 0.4596356927, 0.4912834261, 0.515007608, 0.5347119236,
0.551976436, 0.5585987591,

        0.03381312396, 0.1239420042, 0.1974149358, 0.2661012059, 0.328281334,
0.366202847, 0.4038919979, 0.4464142828, 0.4959539066, 0.5440528846, 0.5840191851,
0.6140868554, 0.6390890295,

        0.07179992369, 0.1404861937, 0.1910017388, 0.2329198819, 0.2726538203,
0.2948676478, 0.3102500263, 0.3234481999, 0.3293037277, 0.3251212079, 0.3269336331,
0.3314879325, 0.3276307198,

        0.0216570545, 0.08466117216, 0.1296581146, 0.1734700098, 0.1990182351,
0.2227191808, 0.2345347993, 0.2480931344, 0.2560399221, 0.2692148595, 0.281762419,
0.2911033799, 0.2991895849

    ]

})


# Polynomial Regression model

degree = 3
```

```
poly = PolynomialFeatures(degree)
X_poly = poly.fit_transform(data[['shear_stress', 'time']])
model = LinearRegression()
model.fit(X_poly, data['surface_concentration'])

# Extract coefficients
coefs = model.coef_
intercept = model.intercept_
features = poly.get_feature_names_out(['shear_stress', 'time'])

# Construct the polynomial equation
terms = [f"{coefs[i]:.6f}*{features[i]}" for i in range(len(coefs))]
equation = " + ".join(terms)
equation = f"{intercept:.6f} + " + equation

# Print R-squared value
r_squared = model.score(X_poly, data['surface_concentration'])
print(f"R-squared value: {r_squared}")

# Plotting
colors = ['blue', 'green', 'red', 'purple', 'orange']
plt.figure(figsize=(12, 8))
for i, stress_level in enumerate(sorted(data['shear_stress'].unique()), start=0):
    # Filter data for each shear stress level
    subset = data[data['shear_stress'] == stress_level]
    # Predict for each subset
    X_subset_poly = poly.transform(subset[['shear_stress', 'time']])
    predictions = model.predict(X_subset_poly)

    # Plot original data
```

```python
    plt.scatter(subset['time'], subset['surface_concentration'], color=colors[i], label=f'Original Data, Tau = {stress_level}')

    # Plot predictions

    plt.plot(subset['time'], predictions, color=colors[i], linestyle='--', label=f'Predicted, Tau = {stress_level}')


plt.title('Comparison of Original and Predicted Surface Concentration by Shear Stress')

plt.xlabel('Time')

plt.ylabel('Surface Concentration')

plt.legend()

plt.grid(True)

plt.show()


print("Polynomial Regression Equation:")

print(f"Surface Concentration = {equation}")

print("Polynomial Regression Equation:")

print(f"Surface Concentration = {equation}")
```

**S3. Residual Plots**: The residual plot helps diagnose the fit of the regression model by showing if residuals are randomly scattered around zero, indicating an unbiased and consistent model.

**S3.1. Model 1** (data with all shear stress 1-5 dyn/cm$^2$):

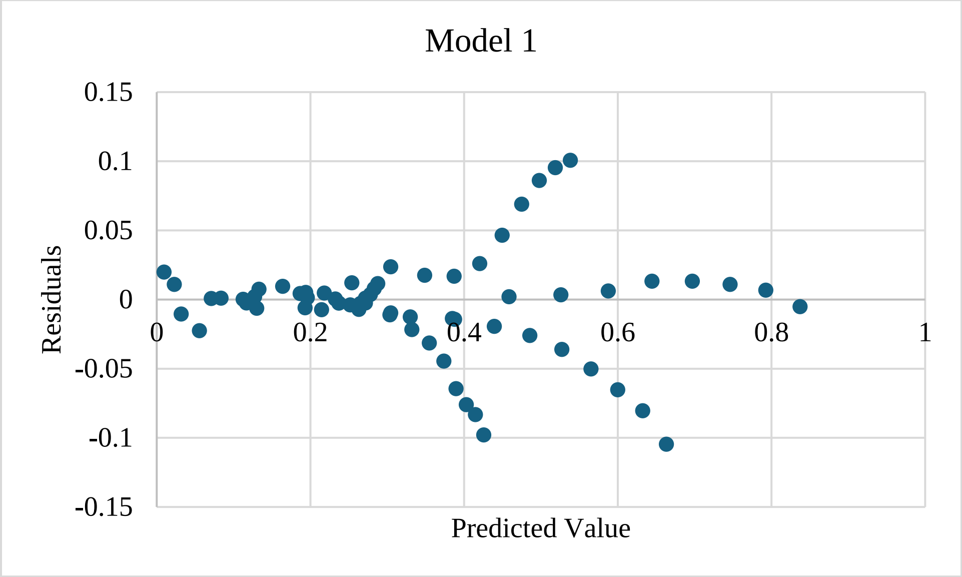


**S3.2. Model 2** (data with only shear stress 1, 2, 4, and 5 dyn/cm$^2$):

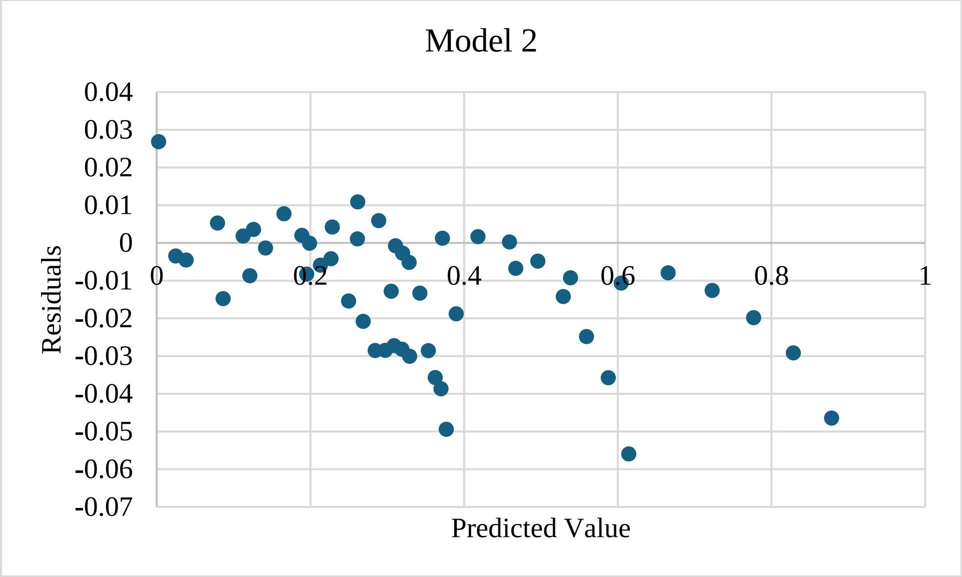

Supplement: Supplementary file 1 [file pathogens-13-00551-s001.zip › pathogens-3076536-supplementary.pdf]
